# Full-Right Full-Left Split Liver Transplantation for Two Adult Recipients: A Single-Center Experience in China

**DOI:** 10.3390/jcm12113782

**Published:** 2023-05-31

**Authors:** Limin Ding, Xizhi Yu, Rui Zhang, Junjie Qian, Wu Zhang, Qinchuan Wu, Lin Zhou, Zhe Yang, Shusen Zheng

**Affiliations:** 1 Division of Hepatobiliary Pancreatic Surgery, First Affiliated Hospital, Zhejiang University School of Medicine, Hangzhou 310003, China; dinglimin@zju.edu.cn (L.D.); 22118092@zju.edu.cn (X.Y.); 12019283@zju.edu.cn (J.Q.); 12033012@zju.edu.cn (Q.W.); 2 NHFPC Key Laboratory of Combined Multi-Organ Transplantation, Hangzhou 310003, China; zhoulin9@zju.edu.cn; 3 Key Laboratory of the Diagnosis and Treatment of Organ Transplantation, CAMS, Hangzhou 310003, China; 4 Key Laboratory of Organ Transplantation, Hangzhou 310003, China; 5 Fuzhou Medical College, Nanchang University, Fuzhou 344000, China; zrui202207@163.com; 6 Department of Hepatobiliary and Pancreatic Surgery, Shulan (Hangzhou) Hospital, Hangzhou 310022, China; zw1116@163.com; 7 Collaborative Innovation Center for Diagnosis Treatment of Infectious Diseases, Hangzhou 310003, China

**Keywords:** split liver transplantation, middle hepatic vein, inferior vena cava, complete splitting and reconstruction

## Abstract

Background: The most effective treatment for end-stage liver diseases is liver transplantation, which is impeded by the shortage of donor livers. Split liver transplantation (SLT) is important for addressing the donor liver shortage. However, full-right full-left SLT for two adult recipients is globally rarely conducted. This study aimed to investigate the clinical outcomes of this technique. Methods: We retrospectively analyzed the clinical data of 22 recipients who underwent full-right full-left SLT at Shulan (Hangzhou) Hospital between January, 2021 and September, 2022. The graft-to-recipient weight ratio (GRWR), cold ischemia time, operation time, length of the anhepatic phase, intraoperative blood loss, and red blood cell transfusion amount were all analyzed. The differences in liver function recovery after transplantation were compared between the left and right hemiliver groups. The postoperative complications and prognosis of the recipients were also analyzed. Results: The livers of 11 donors were transplanted into 22 adult recipients. The GRWR ranged from 1.16–1.65%, the cold ischemia time was 282.86 ± 134.87 min, the operation time was 371.32 ± 75.36 min, the anhepatic phase lasted 60.73 ± 19.00 min, the intraoperative blood loss was 759.09 ± 316.84 mL, and the red blood cell transfusion amount was 695.45 ± 393.67 mL. No significant difference in the levels of liver function markers, total bilirubin, aspartate aminotransferase, or alanine aminotransferase between left and right hemiliver groups at 1, 3, 5, 7, 14, and 28 d postoperatively was observed (both *p* > 0.05). One recipient developed bile leakage 10 d after transplantation, which improved with endoscopic retrograde cholangiopancreatography-guided nasobiliary drainage and stent placement. Another developed portal vein thrombosis 12 d after transplantation and underwent portal vein thrombolytic therapy and stenting to restore portal vein blood flow. A color Doppler ultrasound performed 2 d after transplantation revealed hepatic artery thrombosis in one patient, and thrombolytic therapy was administered to restore hepatic artery blood flow. The liver function of other patients recovered quickly after transplantation. Conclusions: Full-right full-left SLT for two adult patients is an efficient way to increase the donor pool. It is safe and feasible with careful donor and recipient selection. Transplant hospitals with highly experienced surgeons in SLT are recommended to promote using full-right full-left SLT for two adult recipients.

## 1. Introduction

The most effective treatment for end-stage liver diseases is liver transplantation. However, a global shortage of donor livers is impeding the advancement of liver transplantation. Split liver transplantation (SLT) is the process of dividing a donor liver into two or more independent structural and functional grafts for two or more recipients. It is a method used to mitigate the scarcity of donor livers [1]. In clinical practice, donor livers have most commonly been split into the left lateral segment and the right trisegment, or right and left hemilivers, enabling two recipients to receive liver transplants from a single donor and increasing the utilization rate of the liver. The liver is usually divided in SLT into a left lateral segment for a child and a right trisegment for an adult. A donor liver is usually split into two full grafts for two adult recipients. Full-right full-left SLT for two adult recipients is a complex procedure requiring careful recipient and donor selection. Moreover, the allocation of the middle hepatic vein (MHV) has been controversial, and this technique is rarely used at home or abroad. This study aimed to investigate the clinical outcomes of the full-right full-left SLT technique. From January, 2021 to September, 2022, we used 11 donor livers to perform full-right full-left SLT on 22 adult recipients. We describe our experience with this technique in this study.

## 2. Methods and Patients

### 2.1. Ethical Consideration

The study was conducted in accordance with the Declaration of Helsinki (as revised in 2013). Furthermore, the institutional ethics board of Shulan (Hangzhou) Hospital approved the study.

### 2.2. Donor Characteristics

Overall, 11 donors were included. Ten of the 11 donors were classified as China Category I (international standard donation after brain death), and one as China Category III (transitional period of donation after brain death followed by circulatory death). There were ten men and one woman, with a mean age of 41.36 ± 10.49 years and a mean body mass index (BMI) of 23.27 ± 2.37 kg/m^2^. The causes of death were cerebrovascular accidents in five cases, craniocerebral trauma in three cases, and hypoxic-ischemic encephalopathy in three cases. The length of stay in the intensive care unit (ICU) was 4.91 ± 1.92 d. At the time of donation, the total bilirubin (TBIL), aspartate aminotransferase (AST), alanine aminotransferase (ALT), cholinesterase enzyme, and serum sodium levels in donors were 22.36 ± 6.31 μmol/L, 30.55 ± 16.92 U/L, 39.45 ± 17.26 U/L U/L, 4265.09 ± 1866.03 U/L, and 139.00 ± 4.29 mmol/L, respectively. All donors had a stable hemodynamic status, were not on or received low-dose vasopressors, such as dopamine, and had no history of cardiac arrest. Before donation, the liver function of the donors was essentially normal. A liver biopsy using ultrasound guidance revealed no steatosis. Table 1 summarizes the characteristics of the donors.

### 2.3. Recipient Characteristics

Overall, 22 liver transplant recipients were included in the study. There were sixteen men and six women. The mean age of recipients was 50.68 ± 11.98 years, the mean BMI was 22.62 ± 3.67 kg/m^2^, their preoperative Child–Pugh score was 9.91 ± 1.77, and their model for end-stage liver disease (MELD) score was 22.36 ± 11.51. Hepatocellular carcinoma was the primary disease in five cases, intrahepatic cholangiocarcinoma in two, hepatitis B virus (HBV)-related decompensated cirrhosis in eight, decompensated alcoholic cirrhosis in two, and decompensated primary biliary cirrhosis in five. In addition, three recipients had previously undergone a transjugular intrahepatic portosystemic shunt, one underwent splenectomy for splenomegaly with hypersplenism, and one underwent cholecystectomy. The blood types of the donors and recipients were in accordance with the principles of blood transfusion.

### 2.4. Donor Liver Harvest—Splitting Technique

**Figure 1:**
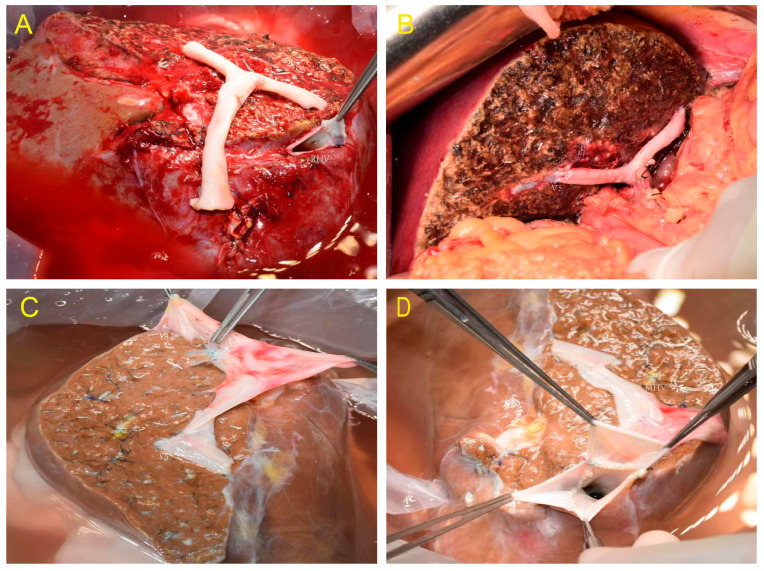
(**A**) The hepatic veins of Segments V and VIII were retained in the right hemiliver and reconstructed with iliac artery. (**B**) No obvious ischemia and congestion were seen after implantation. (**C**) The hepatic veins of Segments V and VIII were retained in the right hemiliver and reconstructed with iliac vein. (**D**) MHV and RHV reconstruction.

Nine out of the eleven donor livers were split in situ. The MHV trunk and vena cava for the left hemiliver and hepatic vein branches in segments V and VIII for the right hemiliver were preserved, followed by angioplasty and reconstruction (Figure 1A–D). Preoperative color Doppler ultrasound was used to assess hepatic steatosis, portal vein, and hepatic vein variations. To assess anatomic variation in the bile ducts, intraoperative cholangiography was performed. The surgical procedure was the same as when harvesting the right hemiliver without the MHV during living donor liver transplantation. The gallbladder was removed, and intraoperative cholangiography was performed through the cystic duct. The right and left hepatic ducts, the main trunk and branches of the hepatic artery, and the right and left branches of the portal vein were separated and exposed after the first hepatic hilum was dissected. The clamping of the right portal vein and the right hepatic artery revealed the right hemihepatic ischemic line. The splitting plane was determined along the MHV-hepatic ischemia line-gallbladder fossa-confluence of right and left hepatic ducts. To expose the MHV, the liver parenchyma was split. The donor liver was perfused, the blood vessels were transected, and the liver was harvested after dissecting the parenchyma. For reconstruction, the MHV was kept for the left hemiliver, and the hepatic veins of Segments V and VIII were kept for the right hemiliver. Furthermore, when small blood vessels and bile ducts were encountered, ligation was performed using titanium clips, silk, or vascular sutures, which were then transected. The hepatic veins of Segments V and VIII had to be reconstructed using iliac vessels from the same donor as much as possible.

**Figure 2:**
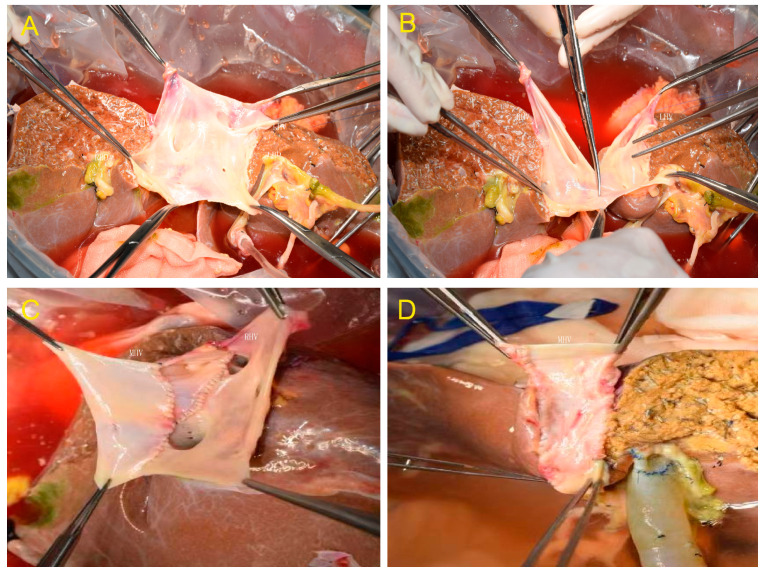
(**A**) After transection of the liver parenchyma and bile duct. (**B**) The retrohepatic inferior vena cava (IVC) was divided by longitudinal transection of the front and back walls. (**C**) Reconstruction of the MHV and IVC in the right hemiliver graft. (**D**) Reconstruction of the MHV and IVC in the left hemiliver graft.

Two out of the eleven donor livers were divided by complete splitting and reconstruction of the MHV and IVC, resulting in a right and left hemiliver graft that included the MHV and IVC ex situ (Figure 2A–D). The hepatic and portal veins were explored after the donor liver, and iliac vessels were harvested using the combined liver–kidney harvesting technique. The volume of the right and left hemilivers was moderate, and cholangiography and hepatic arteriography were performed. The MHV anterior wall was completely dissected and exposed, and the MHV and IVC were split midline. The portal vein, hepatic artery, and bile duct in the first hepatic hilum were transected sequentially, followed by a complete division of the right and left hemilivers. The right hemiliver included the right hepatic duct, the right hepatic artery, and the right branches of the portal vein. The left hemiliver included the common bile duct, the proper hepatic artery, and the main portal vein. The iliac vein from the same donor was used to reconstruct the MHV and IVC in both the left and right hemilivers.

### 2.5. Recipient Surgery

The diseased liver was removed, and the new liver was implanted. Left and right hemiliver grafts were mainly implanted using piggyback or a modified piggyback technique based on the actual matching status of the split donor liver with the recipient. The portal vein, hepatic artery, and bile ducts were reconstructed sequentially. The condition of the bile ducts should be considered when deciding whether to implant a T-tube.

### 2.6. Postoperative Management and Follow-Up

Following transplantation, the following immunosuppressive regimens were used alone or in combination, depending on the condition of the recipients: cyclosporine/tacrolimus/rapamycin, mycophenolate mofetil, and glucocorticoids. Immunosuppressive blood levels were monitored dynamically to maintain stable immunosuppressive blood levels. Routine medication regimens, such as anti-infection, acid-suppressive, liver protection, anticoagulant, anti-HBV, and nutritional support therapies, were also used. Anti-HBV drugs were administered orally, and, if necessary, two antiviral drugs were administered for the anti-HBV treatment. Immune globulin was also administered during hospitalization. Ultrasound examination was used to dynamically monitor liver blood flow and changes in liver function. Following transplantation, all patients were followed up regularly and underwent re-examinations, including routine blood, biochemical, and blood coagulation tests, monitoring of immunosuppressive drug levels in the blood, color Doppler ultrasound of the transplanted liver, abdominal CT, and CT angiography.

### 2.7. Statistical Analysis

The measurement data are expressed as the mean ± SD, and the *t*-test was used to compare groups. All statistical analyses were performed using SPSS software 22.0. Statistical significance was defined as a *p*-value < 0.05.

## 3. Results

The piggyback or modified piggyback technique was used to perform 22 transplants. The mean graft weight was 855.05 ± 152.18 g, the graft-to-recipient weight ratio (GRWR) was 1.16–1.65%, the cold ischemia time was 282.86 ± 134.87 min, and the operation time was 371.32 ± 75.36 min, the length of the anhepatic phase was 60.73 ± 19.00 min, the intraoperative blood loss was 759.09 ± 316.84 mL, and the amount of red blood cell transfusion was 695.45 ± 393.67 mL. Table 2 shows the general characteristics of the recipients in the left and right hemiliver groups. Table 3 shows the status of the liver function recovery after the implantation of the right and left hemilivers. There was no significant difference in the levels of liver function markers (TBIL, AST, or ALT) between the left and right hemiliver groups at 1, 3, 5, 7, 14, and 28 d postoperatively (both *p* > 0.05).

**Figure 3:**
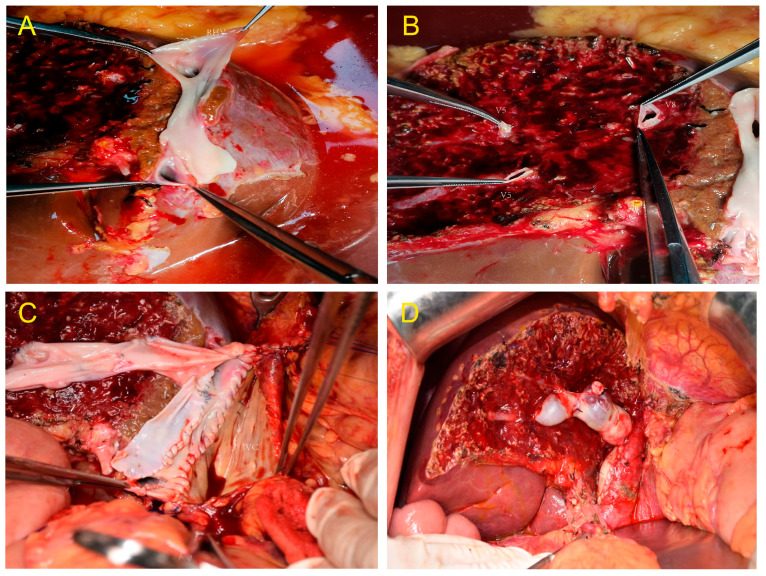
(**A**) Venoplasty of the right hepatic vein and inferior right hepatic vein.(**B**) Segments V and VIII were separately transected during the middle hepatic vein (MHV) splitting. (**C**) Middle hepatic veins (Segments V and VIII) were bridged and reconstructed. (**D**) The MHV drainage of the right hemiliver graft was good, and no obvious ischemia and congestion were noted after implantation.

Two of the eleven donors had Type III portal vein variation. Furthermore, one of the cases was separately transected because the portal vein was H-shaped, and the right anterior and right posterior portal veins were far apart during the splitting. During the MHV splitting, Segments V and VIII were transected and reconstructed with iliac vessels; recipients recovered well after transplantation (Figure 3A–D).

**Figure 4:**
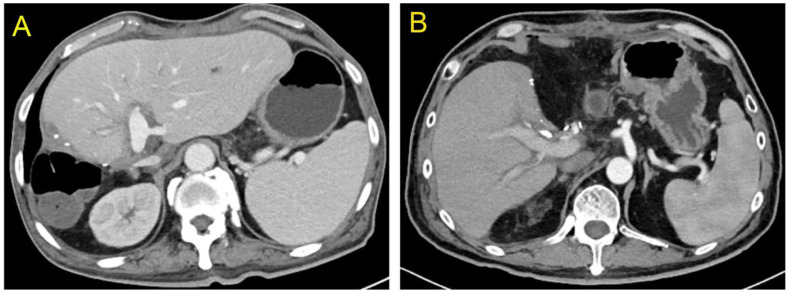
At the time of discharge, no obvious ischemia and congestion were seen in the segments of both the left and right hemilivers near the splitting plane ((**A**) Left hemiliver; (**B**) Right hemiliver).

One of the twenty-two recipients developed bile leakage 10 d after transplantation, which improved with endoscopic retrograde cholangiopancreatography (ERCP)-guided nasobiliary drainage and stent placement. Owing to thrombus formation in the right anterior and right posterior branches of the portal vein 12 d after transplantation, one patient underwent portal vein thrombolytic therapy and stenting, and the portal vein blood flow was then restored. A color Doppler ultrasound performed 2 d after transplantation revealed hepatic artery thrombosis in one recipient. A local filling defect in the hepatic artery and its anastomosis were confirmed with emergency percutaneous super selective digital subtraction angiography. The patient then received catheter-directed thrombolysis. Furthermore, the patient was administered anticoagulation therapy with low-molecular-weight heparin, vasodilator, and microcirculation therapy. The hepatic artery blood flow was restored following treatment. The specifics of post-operative complications and treatments of recipients in left and right hemiliver groups are described in Table 4. The liver function of the remaining recipients recovered quickly after transplantation. Before discharge, a contrast-enhanced abdominal CT examination revealed no obvious ischemia or congestion in the hepatic segments of the left and right hemilivers near the splitting border (Figure 4A,B). All patients were followed up via outpatient visits, and the transplanted livers functioned normally during the follow-up period.

## 4. Discussion

SLT is one of the most common methods for increasing organ utilization; it is used to divide a whole cadaveric donor liver into two or more anatomical functional units based on the principle of liver functional segmentation, allowing for the transplantation of one donor liver into two or more recipients [2,3,4,5,6]. SLT is no longer considered a risk factor affecting liver function recovery, postoperative incidence complications are significantly reduced following SLT, and the efficacy of SLT performed by surgeons in hospitals experienced in this technique is comparable to that of whole liver transplantation [7,8,9,10,11,12,13]. SLT has the potential of increasing the number of donor livers by 15–28% and contributes towards expanding the high-quality donor pool [14,15,16,17,18].

Full-right full-left SLT for two adult recipients is uncommon worldwide, and the quality requirements for the split donor liver are also stringent. Therefore, donor selection criteria differ between transplant centers [19]. The following are the donor selection criteria for SLT: (1) Age < 50 years old, BMI < 26 kg/m^2^, ICU length of stay < 5 d; (2) Hepatic steatosis ratio < 10%, AST/ALT level < 3 times the upper limit of normal, TBIL level < 2 times the upper limit of normal; (3) Serum sodium level < 160 mmol/L, cold ischemia time < 10 h. The following are the recommended recipient selection criteria and the main principles for donor/recipient matching: (1) Adult recipient GRWR > 1.2% and pediatric recipient GRWR of 2–4% [20,21]; (2) No history of complex upper abdominal surgery; (3) Critically ill adult recipients with a MELD score of <30, and critically ill pediatric recipients with a pediatric end-stage liver disease score of <30 [22]. In this study, the mean age of the donors was 41.36 ± 10.49 years, the mean BMI was 23.27 ± 2.37 kg/m^2^, and TBIL, ALT, AST serum levels, and serum sodium in the donors at the time of donation were 22.36 ± 6.31 μmol/L, 30.55± 16.92 U/L, 39.45 ± 17.26 U/L, and 139.00 ± 4.29 mmol/L, respectively. Furthermore, the ICU length of stay was 4.91 ± 1.92 d, indicating that the donors basically met the above-mentioned selection criteria. The GRWR was 1.16% in one of the 22 recipients included in this study, which was higher than the 1.2% in the remaining recipients, and the MELD score was 22.36 ± 11.51. The recipient essentially met the above-mentioned selection criteria. After prompt and active symptomatic treatment, all 22 recipients in this study recovered well. Additionally, no significant difference in the levels of liver function markers, TBIL, AST, and ALT, was found between the left and right hemiliver groups at 1, 3, 5, 7, 14, and 28 d after transplantation.

The allocation of the MHV and IVC in full-right full-left hemiliver splitting depends on the status of the donor and recipient [23,24,25]. The full-right full-left SLT is a complex technique to master. The donor liver can be split in several ways. The allocation of the outflow tracts, i.e., the MHV and IVC allocation, is the key technical point of donor liver splitting. The effective volume of the transplanted liver graft could be ensured by optimal venous drainage. The allocation principle maximizes donor liver utilization. The caudate lobe veins drain directly into the IVC, while Segments IV, V, and VIII drain into the MHV. Patients who received a left hemiliver graft during full-right full-left SLT for two adult recipients had a relatively high risk of developing small-for-size syndrome after transplantation. Venous drainage of Hepatic Segment IV is primarily through the MHV, whereas venous drainage of Hepatic Segment I is primarily through the short hepatic veins. The MHV and IVC were allocated to the left hemiliver in the study of nine donor livers. This has the potential of improving the functional integrity of the left hemiliver grafts [26]. To maximize the use of the donor liver, the hepatic vein branches in Segments V and VIII were retained and reconstructed for the right hemiliver. The GRWR was 1.16–1.65% in recipients included in this study, suggesting that the risk of the small-for-size syndrome was reduced.

Chan et al. [27] believe that donor liver transplantation with a right hemiliver graft without the MHV trunk could produce satisfactory results. Patients who underwent venous reconstruction had a similar prognosis as those who did not undergo vein reconstruction. Gyu et al. [28] also suggested that Segment V and VIII tributaries of the MHV with diameters > 5 mm should be reconstructed and drained into the IVC. Our previous study also revealed that reconstructing the MHV could help patients recover after transplantation by ensuring the patency of the outflow tracts. Hence, the MHV tributaries should be reconstructed as much as possible to expand the hepatic vein anastomosis and reduce liver congestion [29]. However, Fan et al. believe that [30] even if the main branches of the MHV were reconstructed, adequate hepatic venous drainage for the right anterior sector of the graft would still be lacking. This is because the MHV has many branches, and it is impossible to reconstruct all of them. A retained graft with MHV can undoubtedly improve venous drainage during full-right full-left hemiliver splitting. Broering et al. [31] reported a procedure for outflow tract allocation during liver splitting in 2005. The MHV and retrohepatic IVC were split longitudinally and then separately reconstructed using iliac vessels from donors, resulting in full-right full-left hemiliver grafts. This more reasonable procedure allows retaining the MHV drainage of both the left and right hemiliver grafts. The method mentioned above was used in four recipients in this study. The MHV and IVC were split in the midline during the procedure, and many vein orifices on the lateral wall can be seen (Figure 2A–D). The iliac vessels were used to reconstruct the MHV and retrohepatic IVC. This method maximizes the effective volume of the donor liver. In addition, it reduces the degree of graft congestion by preserving the hepatic venous drainage at the transection plane of the left and right hemilivers. These four recipients recovered well after transplantation, with no outflow tract-related complications, biliary complications, or small-for-size syndrome.

The donor portal veins need to be allocated according to the characteristics of the recipient portal vein for full-right full-left liver splitting. The length of the recipient and donor portal veins, the degree of diameter matching, and the reconstruction difficulty should all be considered when deciding whether to keep the portal vein trunk in the left or right hemilivers. Two donors in this study had Type III portal vein variation. We transected them separately because one of the cases was H-shaped, and the right anterior and right posterior portal veins were far apart during the splitting. Additionally, Segments V and VIII were separately transected during the splitting of the MHV and reconstructed with iliac vessels. After transplantation, the blood supply to the left and right hemiliver was good, and the recipients recovered well.

Sneiders et al. and Chan et al. believe that [1,3] full-right full-left SLT for adult recipients may increase the availability of liver grafts, reducing waitlist time. Long-term patient and graft survival appear acceptable and justify transplant benefit in selected patients, which was consistent with our research. One of the twenty-two recipients developed bile leakage 10 d after transplantation, which improved with ERCP-guided nasobiliary drainage and stent placement. One recipient developed portal vein thrombosis 12 d after transplantation. The portal venous blood flow in this patient was restored after undergoing portal vein thrombolytic therapy and stenting. A color Doppler ultrasound performed 2 d after transplantation revealed hepatic artery thrombosis in one recipient, and thrombolytic therapy was used to restore hepatic artery blood flow. Most technical problems encountered with SLT can be solved by strengthening multidisciplinary (such as anesthesiology, artificial liver, ERCP, and angiographic interventions) and multicenter cooperation, improving the level of the SLT technique. No outflow tract-related complications or biliary complications occurred after transplantation. Nonetheless, the sample size of this study was small; hence, large-scale studies are needed to evaluate the efficacy of the MHV allocation procedure used in this study. Furthermore, the follow-up period in this study was relatively brief. In the future, we will continue to promote the use of full-right full-left SLT for two adult recipients and further strengthen follow-up and efficacy evaluation.

## Figures and Tables

**Table 1:** Donor characteristics.

Groups	Values
Age (year)	41.36 ± 10.49
Height (cm)	171.27 ± 4.50
Weight (kg)	68.18 ± 6.46
BMI (kg/m^2^)	23.27 ± 2.37
TBIL (μmol/L)	22.36 ± 6.31
ALT (U/L)	30.55 ± 16.92
AST (U/L)	39.45 ± 17.26
Cholinesterase (U/L)	4265.09 ± 1866.03
Serum Sodium (mmol/L)	139.00 ± 4.29
ICU Stay (days)	4.91 ± 1.92

BMI, body mass index; TBIL, total bilirubin; ALT, alanine aminotransferase; AST, aspartate aminotransferase; ICU, intensive care unit.

**Table 2:** Characteristics of recipients in left and right hemiliver groups.

Groups	Left Hemiliver Group	Right Hemiliver Group
Age (year)	51.91 ± 13.00	49.45 ± 11.36
Height (cm)	166.55 ± 6.79	169.18 ± 6.91
Weight (kg)	60.14 ± 9.14	68.36 ± 11.20
BMI (kg/m^2^)	21.76 ± 3.70	23.48 ± 3.59
Child–Pugh score	9.91 ± 1.92	9.91 ± 1.70
MELD score	22.73 ± 11.83	22.00 ± 11.75
Weight of the graft (g)	771.55 ± 102.89	938.55 ± 150.69
GRWR (%)	1.29 ± 0.09	1.38 ± 0.15
Cold ischemia time (min)	268.55 ± 115.08	297.18 ± 156.54
Operation time (min)	346.64 ± 54.04	396.00 ± 87.55
Length of the anhepatic phase (min)	53.00 ± 13.51	68.45 ± 21.08
Intraoperative blood loss (mL)	650.00 ± 242.90	868.18 ± 354.45
Amount of red blood cell transfusion (mL)	554.55 ± 343.11	836.36 ± 405.03

BMI, body mass index; MELD score, the model for end-stage liver disease score; GRWR, graft-to-recipient weight ratio.

**Table 3:** Liver function status of the recipients after full-right full-left split liver transplantation.

Days after Surgery	Left Hemiliver Group (n = 11)			Right Hemiliver Group (n = 11)		
	TBIL (μmol/L)	ALT (U/L)	AST (U/L)	TBIL (μmol/L)	ALT (U/L)	AST (U/L)
1	178.10 ± 100.12	429.10 ± 355.66	887.00 ± 848.65	131.80 ± 91.30	683.60 ± 455.01	862.20 ± 561.63
3	136.40 ± 90.91	243.70 ± 168.10	211.20 ± 164.56	111.30 ± 88.90	492.30 ± 376.72	279.50 ± 221.27
5	105.70 ± 75.93	132.30 ± 90.69	61.30 ± 32.48	68.30 ± 27.97	204.10 ± 153.91	59.20 ± 30.76
7	102.60 ± 70.45	66.70 ± 28.79	45.90 ± 24.24	64.10 ± 28.80	126.70 ± 95.04	45.80 ± 22.04
14	84.20 ± 73.36	58.70 ± 32.70	62.40 ± 49.57	54.90 ± 28.98	53.10 ± 32.76	34.60 ± 11.10
28	58.70 ± 55.92	41.20 ± 18.68	43.60 ± 24.11	34.00 ± 27.19	35.80 ± 26.83	27.40 ± 11.16

Data are expressed as mean ± SD. TBIL, total bilirubin; ALT, alanine aminotransferase; AST, aspartate aminotransferase.

**Table 4:** Post-operative complications and treatments of recipients in left and right hemiliver groups.

Recipients	Grafts	Post-Operative Complications	Days after Surgery	Treatments
1	Right hemiliver	Bile leakage	10 days	Nasobiliary drainage and stent placement.
2	Left hemiliver	Favorable prognosis	-	-
3	Right hemiliver	Favorable prognosis	-	-
4	Left hemiliver	Favorable prognosis	-	-
5	Right hemiliver	Favorable prognosis	-	-
6	Left hemiliver	Favorable prognosis	-	-
7	Left hemiliver	Favorable prognosis	-	-
8	Right hemiliver	Favorable prognosis	-	-
9	Left hemiliver (MHV-IVC *)	Favorable prognosis	-	-
10	Right hemiliver (MHV-IVC *)	Favorable prognosis	-	-
11	Right hemiliver (MHV-IVC *)	Favorable prognosis	-	-
12	Left hemiliver (MHV-IVC *)	Favorable prognosis	-	-
13	Left hemiliver	Portal vein thrombosis	12 days	Portal vein thrombolytic therapy and stenting
14	Right hemiliver	Favorable prognosis	-	-
15	Right hemiliver	Favorable prognosis	-	-
16	Left hemiliver	Favorable prognosis	-	-
17	Right hemiliver	Favorable prognosis	-	-
18	Left hemiliver	Favorable prognosis	-	-
19	Left hemiliver	Favorable prognosis	-	-
20	Right hemiliver	Favorable prognosis	-	-
21	Right hemiliver	Hepatic artery thrombosis	2 days	Thrombolytic therapy
22	Left hemiliver	Favorable prognosis	-	-

* MHV-IVC, MHV and retrohepatic IVC were split longitudinally and then separately reconstructed using iliac vessels from donors.

## Data Availability

The data presented in this study are available on request from the corresponding author.

## Abbreviations

SLT	Split liver transplantation
GRWR	Graft-to-recipient weight ratio
MHV	Middle hepatic vein
BMI	Body mass index
ICU	Intensive care unit
TBIL	Total bilirubin
AST	Aspartate aminotransferase
ALT	Alanine aminotransferase
MELD	Model for end-stage liver disease
HBV	Hepatitis B virus
IVC	Inferior vena cava
ERCP	Endoscopic retrograde cholangiopancreatography
RBD	Right bile duct
LBD	Left bile duct
V5	Liver segment 5
V8	Liver segment 8
RHV	Right hepatic vein
LHV	Left hepatic vein
RAP	Right anterior portal vein
RPP	Right posterior portal vein
S5	Venae hepatis 5th stage of reflux
S8	Venae hepatis 8th stage of reflux
PV	Portal vein
MHV-IVC	MHV and retrohepatic IVC were split longitudinally and then separately reconstructed using iliac vessels from donors
