# Correction: Ding et al. Full-Right Full-Left Split Liver Transplantation for Two Adult Recipients: A Single-Center Experience in China. *J. Clin. Med.* 2023, *12*, 3782

**DOI:** 10.3390/jcm12185991

**Published:** 2023-09-15

**Authors:** Limin Ding, Xizhi Yu, Rui Zhang, Junjie Qian, Wu Zhang, Qinchuan Wu, Lin Zhou, Zhe Yang, Shusen Zheng

**Affiliations:** 1 Division of Hepatobiliary Pancreatic Surgery, First Affiliated Hospital, Zhejiang University School of Medicine, Hangzhou 310003, China; dinglimin@zju.edu.cn (L.D.);; 2 NHFPC Key Laboratory of Combined Multi-Organ Transplantation, Hangzhou 310003, China; 3 Key Laboratory of the Diagnosis and Treatment of Organ Transplantation, CAMS, Hangzhou 310003, China; 4 Key Laboratory of Organ Transplantation, Hangzhou 310003, China; 5 Fuzhou Medical College, Nanchang University, Fuzhou 344000, China; 6 Department of Hepatobiliary and Pancreatic Surgery, Shulan (Hangzhou) Hospital, Hangzhou 310022, China; 7 Collaborative Innovation Center for Diagnosis Treatment of Infectious Diseases, Hangzhou 310003, China

## Error in Figure/Table

In the original publication [1], there was a mistake in Table 1, Figure 1, Figure 2 and Figure 3 as published. The previous description was not sufficient and clear, in order to more accurately describe the operation and treatment, the corrected Table 1, Figure 1, Figure 2 and Figure 3 are shown below. 

## Text Correction

There were several errors in the original publication.

1. “thrombectomy” was corrected to “thrombolytic therapy” in the original article where it appeared. The correction has been made to Abstract; Results, Paragraph 3; Discussion, Paragraph 6; and Table 4.
2. There was only one patient with H type of portal vein, the original article was corrected where it appeared. The correction has been made to Results, Paragraph 2; and Discussion, Paragraph 5.
3. Figure serial number changed and deleted. The correction has been made to Section 2.4, the “Figure 1A,B” should be updated to “Figure 1A–D”; and Section 2.5, the “Figure 2E,F” should be deleted.

The authors state that the scientific conclusions are unaffected. This correction was approved by the Academic Editor. The original publication has also been updated.

## Figures and Tables

**Figure 1:**
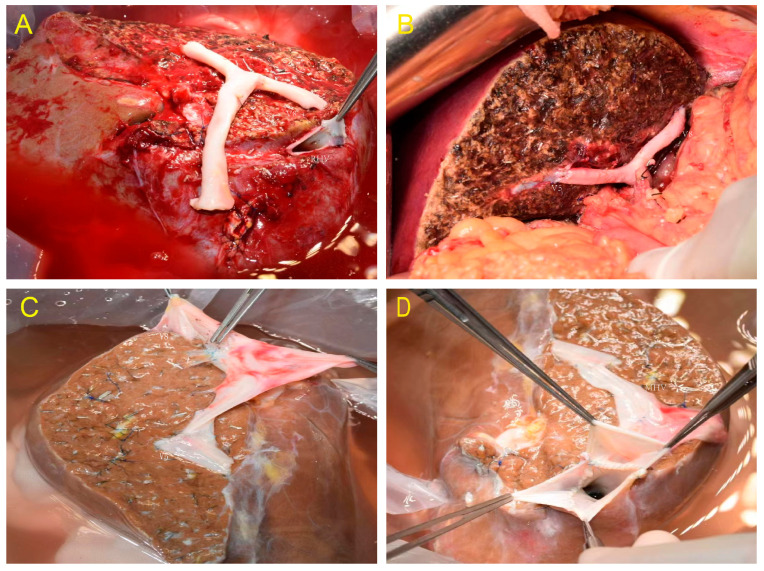
(**A**) The hepatic veins of Segments V and VIII were retained in the right hemiliver and reconstructed with iliac artery. (**B**) No obvious ischemia and congestion were seen after implantation. (**C**) The hepatic veins of Segments V and VIII were retained in the right hemiliver and reconstructed with iliac vein. (**D**) MHV and RHV reconstruction.

**Figure 2:**
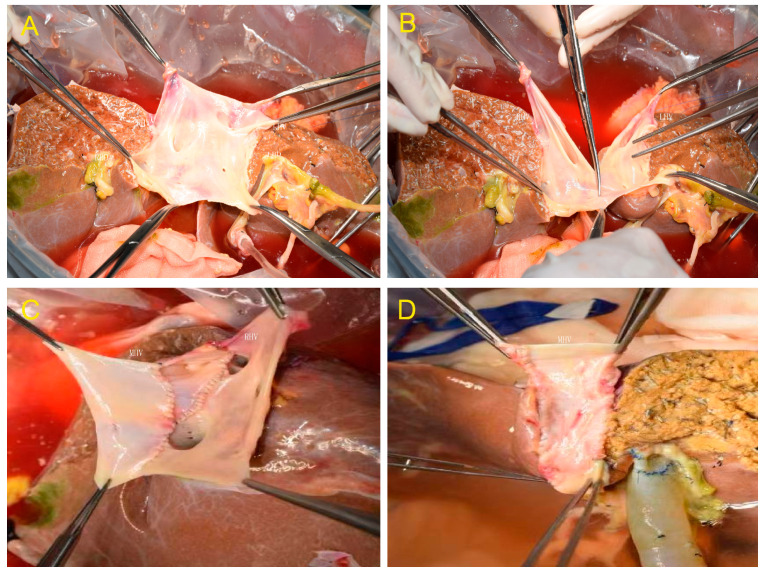
(**A**) After transection of the liver parenchyma and bile duct. (**B**) The retrohepatic inferior vena cava (IVC) was divided by longitudinal transection of the front and back walls. (**C**) Reconstruction of the MHV and IVC in the right hemiliver graft. (**D**) Reconstruction of the MHV and IVC in the left hemiliver graft.

**Figure 3:**
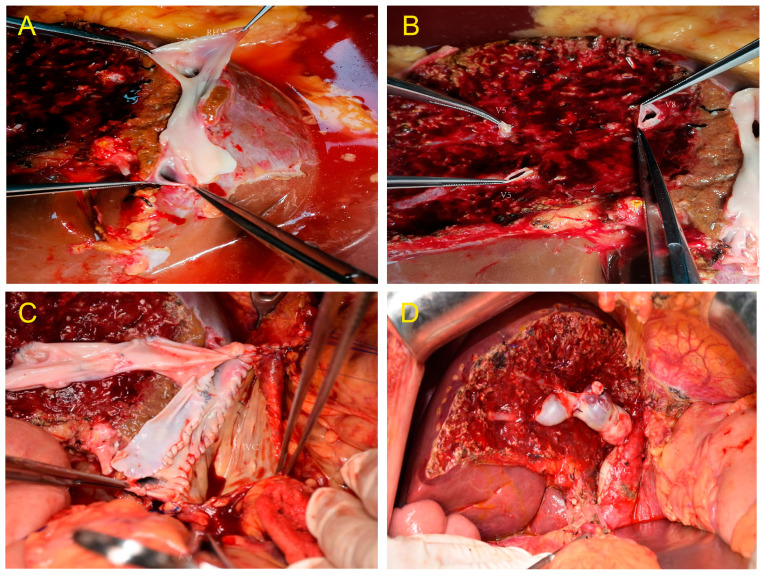
(**A**) Venoplasty of the right hepatic vein and inferior right hepatic vein.(**B**) Segments V and VIII were separately transected during the middle hepatic vein (MHV) splitting. (**C**) Middle hepatic veins (Segments V and VIII) were bridged and reconstructed. (**D**) The MHV drainage of the right hemiliver graft was good, and no obvious ischemia and congestion were noted after implantation.

**Table 1:** Donor characteristics.

Groups	Values
Age (year)	41.36 ± 10.49
Height (cm)	171.27 ± 4.50
Weight (kg)	68.18 ± 6.46
BMI (kg/m^2^)	23.27 ± 2.37
TBIL (μmol/L)	22.36 ± 6.31
ALT (U/L)	30.55 ± 16.92
AST (U/L)	39.45 ± 17.26
Cholinesterase (U/L)	4265.09 ± 1866.03
Serum Sodium (mmol/L)	139.00 ± 4.29
ICU Stay (days)	4.91 ± 1.92

BMI, body mass index; TBIL, total bilirubin; ALT, alanine aminotransferase; AST, aspartate aminotransferase; ICU, intensive care unit.
